# FIB-4 Index Can Predict Mortality in Hospitalized Patients with COVID-19 Infection, Independent of CT Severity Score

**DOI:** 10.34172/aim.33514

**Published:** 2025-02-01

**Authors:** Faeze Salahshour, Sahar Karimpour Reyhan, Kazem Zendedel, Kiana Seifouri, Monireh Sadat Seyyedsalehi, Parnian Naghavi, Mahsa Abbaszadeh, Alireza Esteghamati, Manouchehr Nakhjavani, Soghra Rabizadeh

**Affiliations:** ^1^Department of Radiology, Advanced Diagnostic and Interventional Radiology Research Center (ADIR), Tehran University of Medical Sciences, Tehran, Iran; ^2^Endocrinology and Metabolism Research Center (EMRC), Vali-Asr Hospital, Tehran University of Medical Sciences, Tehran, Iran; ^3^Cancer Research Center, Cancer Institute, Tehran University of Medical Sciences, Tehran, Iran; ^4^Department of Medical and Surgical Sciences, University of Bologna, 40138 Bologna, Italy; ^5^Department of Information Engineering, University of Padua, Padua, Italy

**Keywords:** COVID-19, FIB-4 index, Infectious disease, Mortality rate

## Abstract

**Background:**

The fibrosis 4 (FIB-4) index is typically used in assessing liver fibrosis, and has shown potential in predicting the outcome in various diseases. This study aims to evaluate the predictive power of the FIB-4 index for mortality in COVID-19 patients admitted to a reference hospital in Tehran, Iran.

**Methods:**

In this prospective cohort study, 387 patients with COVID-19 without diabetes, were categorized into deceased and surviving groups. We compared anthropometric and demographic data, liver function tests, CT scores, and FIB-4 indices between the groups. Multivariate logistic regression assessed the independent association of FIB-4 with mortality.

**Results:**

Among the 387 patients, (all non-diabetics), 58 (15%) died, with a higher mortality rate observed in patients with a FIB-4 index≥2.6 (63.4%) compared to those with FIB-4<2.6 (29.7%). Deceased patients were considerably older and more likely to be hypertensive (*P* values<0.001). After adjustment of confounding factors, a FIB-4 index≥2.6 was found to be independently associated with increased mortality (OR: 13.511, 95% CI: 1.356-134.580, *P*=0.026).

**Conclusion:**

The FIB-4 index, calculable by routine laboratory tests, may be a valuable prognostic factor for COVID-19 mortality. This easily obtainable marker could help identify high-risk patients early, potentially allowing for more rapid intervention and treatment prioritization.

## Introduction

 The COVID-19 pandemic caused by human-to-human transmission of SARS-CoV-2, has led to millions of deaths worldwide since its rapid spread began in March 2020.^1,2^ Although most individuals infected with COVID-19 experience mild to moderate symptoms, some patients develop more severe disease, resulting in hospitalization and mortality.^3^ Researchers have actively sought to identify risk factors for disease severity and mortality as the outbreak persists. Such factors could help clinicians prioritize high-risk patients for early admission and targeted interventions.^4^

 Several risk factors for severe COVID-19 and mortality have been identified, including older age, male gender, underlying medical conditions such as cancer, immune deficiency, diabetes, hypertension, cardiovascular disorders, chronic pulmonary disorders, electrolyte imbalances, depression, and obesity. However, there is still limited knowledge about the factors that link to mortality.^5-7^

 COVID-19 can cause liver injury for multiple reasons including direct viral damage, systematic inflammatory reactions, drug-induced, and ischemia-reperfusion damage. It is noteworthy to mention that the virus attaches to the angiotensin-converting enzyme 2 (ACE-2) receptor that leads to liver dysfunction and hepatobiliary injury.^8^ The fibrosis 4 (FIB-4) index is a non-invasive biomarker initially developed to estimate liver fibrosis. It is calculated by using the patient’s age, alanine aminotransferase (ALT), aspartate aminotransferase (AST), and platelet count.^9^ A FIB-4 score ≥ 2.6 indicates advanced liver fibrosis and patients who have FIB-4 < 1.3 are at low risk for liver fibrosis.^10,11^ The FIB-4 index has been demonstrated to have predictive value for liver complications and mortality in patients with chronic liver disease and diabetes.^12^ Recent studies have suggested that the FIB-4 index may also be linked with COVID-19 mortality.^13^ However, these studies are limited by small sample sizes, lack of multivariable adjustment, inconsistent findings, and lack of information about underlying liver dysfunction for adjusting analysis, highlighting the need for further investigation.^14^ The potential role of the FIB-4 index as a predictor of mortality in COVID-19 patients remains uncertain and requires further research.^15^ However, it is believed one of the reasons is that when decompensation happens, it can lead to liver failure and multi-organ dysfunction.^16^

 The FIB-4 score can be easily calculated using routine laboratory tests. If validated as a predictor of COVID-19 severity, it could aid in the early identification of high-risk patients, allowing for the potential implementation of more intensive interventions. This study aims to evaluate the FIB-4 index as a potential easy-to-use and cost-effective predictor of disease severity and mortality in COVID-19 patients, which could complement or precede more resource-intensive evaluation such as imaging.

## Materials and Methods

###  Study Population

 This prospective cohort study was conducted at Tehran University of Medical Science, including all COVID-19 patients admitted to Vali-Asr Hospital in 2020. The patients were followed from the time of admission to the hospital until the time of discharge or death.

 The Tehran University of Medical Sciences Board of Ethics endorsed the study protocol with the ethical code of IR.TUMS.IKHC.REC.1398.1038. Written informed consent was obtained from all study participants or their legal representatives. The study was conducted according to the Declaration of Helsinki.

 The inclusion criteria were all patients who were hospitalized with COVID-19 during 2020. The exclusion criteria were patients with diabetes, cirrhosis, and a history of liver disease to avoid confounding effects on the FIB-4 index. Given that diabetes mellitus is known to independently elevate the FIB-4 index, we excluded patients with diabetes from this study to avoid confounding effects. A total of 387 patients met the criteria and were included in the study. The main outcome of this study was in-hospital mortality rate.

###  Data Collection 

 The data collected from patients’ medical records encompasses a range of demographic and clinical characteristics, laboratory measurements, and relevant medical histories. The demographic and clinical data include age, gender, weight, height, body mass index (BMI), systolic and diastolic blood pressure, body temperature, oxygen saturation, pulse rate, respiratory rate, smoking history, current medications, and significant medical histories such as cardiovascular disease (CVD), chronic kidney disease (CKD), and cancer.

 Laboratory measurements cover various diagnostic tests: fasting blood glucose (FBS), liver function tests (including ALT, AST, and ALP), electrolyte levels (calcium, phosphorus, magnesium), renal function tests (urea and creatinine), complete blood count (hemoglobin and platelet count), erythrocyte sedimentation rate (ESR), and serum albumin. Height was measured to the nearest centimeter using a standard height board, with participants standing in an upright position. Weight was measured to the nearest 0.1 kg using a calibrated digital scale.

 BMI was calculated as weight (kg) divided by height squared (m^2^). Blood pressure was measured using a standard sphygmomanometer (Riester, Big Ben adults, Germany). Two readings were taken at 10-minute intervals after the participant had rested for at least 10 minutes in a seated position. Blood samples were collected after a 12-hour overnight fast and analyzed using standard laboratory kits. FBS was assessed using a glucose oxidase test with enzymatic calorimetry methods. COVID-19 was provisionally diagnosed based on clinical features and symptoms and confirmed by a positive reverse transcription polymerase chain reaction (RT-PCR) nasopharyngeal swab test.^17^ The admission FIB-4 index was measured (age [years] × AST [U/L] / [platelet count (10^9^/L) × ALT (U/L)^1/2^]) and classified as < 2.6 or ≥ 2.6 according to previous studies.^10,11^

 Lung involvement in COVID-19 was assessed using computed tomography (CT) scans, which can reveal three primary patterns: ground-glass opacity, lung consolidation, or a combination of both. These patterns were evaluated across the different lung lobes: upper, middle, and lower lobes of the right lung, and upper and lower lobes of the left lung. An expert radiologist scored the severity of involvement for each lobe based on the pattern and extent of involvement. The scores were aggregated to yield a total score ranging from 0 to 25. The total CT scores were categorized into six grades of lung involvement severity as follows: Grade 0 (score 0, no involvement), Grade 1 (scores 0-5), Grade 2 (scores 5-10), Grade 3 (scores10-15), Grade 4 (scores 15-20), and Grade 5 (scores 20-25) (Table 1).

**Table 1:** Lung Involvement Calculation.

**Lung Involvement Calculation**	**Score **
Right Upper Lobe Ground Glass + Right Upper Lobe Consolidation = Right Upper Lobe total	0-5
Right Middle Lobe Ground Glass + Right Middle Lobe Consolidation = Right Middle Lobe total	0-5
Right Lower Lobe Ground Glass + Right Lower Lobe Consolidation = Right Lower Lobe total	0-5
Left Upper Lobe Ground Glass + Left Upper Lobe Consolidation = Left Upper Lobe total	0-5
Left Lower Lobe Ground Glass + Left Lower Lobe Consolidation = Left Lower Lobe total	0-5
Total Score = Right Upper Lobe total + Right Middle Lobe total + Right Lower Lobe total + Left Upper Lobe total + Left Lower Lobe total	0-25

###  Statistical Analysis

 Data analysis was performed using SPSS version 24 (IBM Corp., Armonk, NY, USA). For continuous variables, data with normal distribution were reported as mean ± standard deviation, and as median and interquartile range for data without normal distribution. All continuous variables were compared between two groups of surviving and deceased patients using a* t* test. For categorical variables, frequency (percentage) was reported compared using chi-square test.

 To assess the independent relationship between the FIB-4 index and COVID-19 mortality, we performed a multivariate logistic regression analysis. The model was adjusted for potential confounding factors such as age, gender, calcium levels, lymphocyte count, hemoglobin, CVD, CKD, cancer, and CT severity score. Results are presented as odds ratios (ORs) with 95% confidence intervals (CIs). Statistical significance was described as a *P *value less than 0.05.

## Results

 Among the 387 patients with COVID-19 (without T2D), 58 (15%) died from the infection during hospitalization.

Table 1 summarizes the baseline characteristics of the study population. Patients in the deceased group were significantly older than those in the surviving group (68.09 ± 12.82 years vs. 61.95 ± 12.34 years, *P* = 0.001). Gender distribution analysis revealed that 16 of 145 (11.0%) female patients and 42 of 242 (17.4%) male patients died (*P* = 0.06). In comparison with the surviving group, patients in the deceased group were more likely to be hypertensive and older (*P* < 0.001). While the difference in mortality between the genders approached statistical significance (*P* = 0.06), it was not statistically significant and should be interpreted with caution.

 History of smoking was higher in the death group (*P* = 0.56). These patients had higher rates of CVD, CKD, and cancer in their past medical history (*P* values = 0.004, 0.001, 0.01, respectively). However, it is worth mentioning that the limited sample size can be the reason for significantly higher cases of cancer in our study. Also, liver enzymes (ALT (IU/L), AST (IU/L), ALP (IU/L)), phosphorus (mg/dL), urea (mg/dL), Cr (mg/dL), CT score, and Mg (mg/dL) were significantly higher in this group (*P* < 0.05). In these patients, white blood cell count was significantly increased (*P* < 0.001), mainly due to elevated neutrophil counts. Conversely, level of calcium (mg/dL), hemoglobin (g/dL), albumin(g/dL), and lymphocyte counts were significantly lower in the deceased group (all *P* < 0.05). These patients had higher levels of FIB-4 (*P* = 0.002). The number of patients having FIB-4 higher than 2.6 was significantly higher in these patients. (Table 2) The multivariable logistic regression model showed a significant independent relationship between FIB-4 and mortality. This association remained significant after adjusting for potential confounding factors including age, gender, calcium, lymphocyte count, Hb, CVD, CKD, cancer, and CT score total. The odd ratio for the FIB-4 index was found to be 13.511(*P* value = 0.026, 95% CI: 1.356 -134.580) (Table 3).

**Table 2:** Comparison of Clinical Features Between the Deceased and Surviving Groups

**Basal Characteristics**	**Death Group**	**Surviving Group**	***P*** ** Value **
Age	68.09 ± 12.82	61.95 ± 12.34	0.001
Gender (F/M)	16/42	129/200	0.06
Smoking %	8.6	6.4	0.56
CVD %	29.3	14	0.004
CKD %	13.8	3.6	0.001
Cancer %	12.1	4	0.01
FIB-2.6 %	63.4	29.7	0.00
weight (kg)	74.85 ± 17.04	78.88 ± 15.25	0.357
Height (cm)	171.22 ± 9.77	167.80 ± 9.68	0.741
BMI (kg/m^2^)	25.44 ± 4.72	27.86 ± 4.88	0.019
Systolic BP (mm Hg)	124.16 ± 20.46	122.01 ± 16.76	0.42
Diastolic BP (mm Hg)	75.45 ± 15.04	76.23 ± 12.23	0.69
RR (/min)	27.5 ± 13.54	21.33 ± 7.16	0.00
PR (beat/min)	97.31 ± 17.82	93.93 ± 17.54	0.20
Temperature (centigrade)	37.43 ± 0.89	37.45 ± 0.93	0.86
Oxygen saturation(%)	82.28 ± 13.17	90.23 ± 5.51	0.00
FBS (mg/dL)	155.94 ± 85.24	127.4 ± 55.46	0.06
ALT (IU/L)	46.61 ± 34.67	37.03 ± 25.10	0.008
AST (IU/L)	53.44 ± 34.08	42.34 ± 28.28	0.008
ALP (IU/L)	214.21 ± 132.30	175.74 ± 89.43	0.019
Calcium (mg/dL)	8.05 ± 0.68	8.35 ± 0.66	0.013
phosphorus (mg/dL)	3.92 ± 1.57	3.34 ± 1.11	0.011
Urea (mg/dL)	84.04 ± 68.75	36.39 ± 21.51	0.00
Cr (mg/dL)	2.16 ± 1.20	1.93 ± 0.87	0.00
ESR (mm/h)	65.28 ± 36.44	62.25 ± 31.91	0.55
Hb (g/dL)	12.60 ± 2.01	13.30 ± 2.01	0.024
WBC (per μL)	9.62 ± 3.90	6.89 ± 3.25	0.00
Neutrophil %	83.32 ± 13.58	74.06 ± 11.72	0.00
Lymph %	12.69 ± 11.91	20.13 ± 10.08	0.00
PLT (per μL)	212.02 ± 81.22	219.23 ± 93.97	0.61
Albumin (g/dL)	3.04 ± 0.59	3.65 ± 0.53	0.00
Mg (mg/dL)	2.17 ± 0.33	2.06 ± 0.34	0.06
FIB-4	3.66 ± 2.78	2.41 ± 2.18	0.002
CT severity score total	13.67 ± 6.95	8.55 ± 5.15	< 0.001

CVD %: cardiovascular disease, CKD %: chronic kidney disease, FIB-2.6 %: FIB-4 index more than 2.6, BMI: Body mass index, Systolic BP: Systolic blood pressure, Diastolic BP: Diastolic blood pressure, RR: Respiratory Rate, PR: pulse Rate, FBS: Fasting Blood Sugar, ALT: alanine aminotransaminase, AST: Aspartate aminotransferase, ALP: Alkaline phosphatase, Cr: creatinine, ESR: Erythrocyte sedimentation rate, Hb: hemoglobin, WBC: White Blood Cell, PLT: platelet, Mg: magnesium. Data are presented as mean ± standard deviation.

**Table 3:** Results of Multivariate Logistic Regression

	**Beta **	**Standard error**	**Odds ratio**	**95% CI**		***P*** ** Value**
				**Lower**	**Upper**	
FIB-2.6	2.603	1.173	**13.511**	1.356	134.580	0.026
Gender	2.892	2.892	18.034	0.952	341.737	0.054
Age	0.019	0.040	1.020	0.943	1.102	0.626
Calcium	-1.169	0.953	0.311	0.048	2.011	0.220
Lymph	-0.086	0.050	0.918	0.832	1.012	0.085
Hb	-0.178	0.353	0.837	0.419	1.671	0.837
CVD	2.083	1.165	8.032	0.820	78.714	0.074
CKD	-3.248	2.805	0.039	0.000	9.472	0.274
Cancers	-20.032	18550.415	0.000	0.000		0.999
CT severity total score	0.072	0.077	1.075	0.924	1.250	0.350

CVD: cardiovascular disease, CKD: chronic kidney disease, FIB-2.6: FIB-4 index more than 2.6.

## Discussion

 This study investigated the association between the FIB-4 index and COVID-19 mortality. Multivariable logistic regression analysis, adjusted for potential confounders including CT scan severity score, revealed that patients with a higher FIB-4 index were 13.5 times more at risk of having severe COVID-19 which can lead to death (OR: 13.5, 95% CI: 1.356-134.580). The FIB-4 index demonstrated an independent association with COVID-19 mortality, even after controlling for other known risk factors. All hospitalized COVID-19 patients were included in the study and there was not any selection bias in the study.

 COVID-19 is an inflammatory disease that can cause hyperactivity of immune cells followed by hypersecretion of cytokines, potentially resulting in elevated liver enzymes such as ALT and AST.^18,19^ Additionally, drug-induced liver injury (DILI) from antibacterial, antiviral, and vasopressor treatments in severe cases may be a contributing factor to increased liver enzymes.^20^ Under these circumstances, numerous organs including the liver may suffer detrimental effects. Consequently, the observed increase in the FIB-4 index among our hospitalized COVID-19 patients was anticipated. Previous studies have demonstrated a correlation between FIB-4 index and COVID-19 severity. In a cohort of non-alcoholic fatty liver disease (NAFLD) patients, the level of FIB-4 index correlated with the severity of COVID-19.^21^ However, the increase in the FIB-4 index may be due to underlying liver disease. A recent study suggested that FIB-4 has specific properties to evaluate outcomes unrelated to fibrosis or NAFLD.^22^ In a study by Rentsch et al, of 585 patients with positive COVID-19, 297 were hospitalized. Among these, 122 needed ICU care. After adjusting for DM, renal, cardiovascular, and respiratory diseases, FIB-4 > 3.25 had an OR of 8.73 (95% CI, 4.11 -18.56) for hospitalization and an OR of 8.40 (95% CI, 2.90-24.28) for ICU admission in comparison with those who had FIB-4 < 1.45.^23^ Also in our study, after excluding patients with diabetes and adjusting for significant clinical diseases (renal and CVD, and cancer), the FIB-4 index was observed as an independent factor for mortality in patients with COVID-19. These findings are consistent with other recent studies. In a Spanish cohort of 160 hospitalized COVID-19 patients, Ibáñez-Samaniego et al found that a FIB-4 index > 2.67 (OR: 3.42, 95% CI: 1.30-8.92) was associated with increased ICU admission.^24^ Sterling et al corroborated this relationship, noting no significant difference in the prevalence of underlying liver disease between patients requiring ventilator support and those who did not.^25^ However, our study observed a significantly higher prevalence of CKD in the deceased group.

 Further supporting these findings, a Romanian study of 138 patients with type 2 diabetes demonstrated that higher FIB-4 indices correlated with more severe disease, increased ICU admission, and higher mortality rates.^26^ Meta-analyses have strengthened this evidence base. Pranata et al reported that FIB-4 predicted severe COVID-19 outcomes with 56% sensitivity and 80% specificity,^27^ while Liu et al demonstrated a positive linear correlation between FIB-4 and both severe COVID-19 and mortality.^28^ Liver dysfunction is common in coronavirus infection,^29^ and our findings align with previous studies indicating that liver injury is associated with higher mortality rates in COVID-19 patients.^30^ Within this context, it can be assumed that high-grade fibrosis may accelerate the risk of developing an aggravated inflammatory response, a characteristic feature of progressive COVID-19. Genuinely, advanced liver fibrosis is illustrated by the constant activation of immune system cells by pathogen-associated molecular patterns (PAMPs) and damage-associated molecular patterns (DAMPs) that enhance the release of cytokines and chemokines which are precursors of developing inflammation.^31,32^ Ibáñez-Samaniego et al showed a positive correlation between FIB-4 and CRP that further supports the mentioned hypothesis.^24^

 The FIB-4 index is a non-invasive tool for assessing liver fibrosis in patients with type 2 diabetes. It is calculated using age, AST, ALT, and platelet count. A higher FIB-4 level indicates more advanced liver fibrosis.^12,33^ While the precise mechanism underlying this relationship is not fully elucidated, transient immune liver injury, ACE2/ DPP4-mediated hepatocyte injury, ischemia, hypoxia, thrombosis, and drug hepatotoxicity have been proposed as potential contributors.^13^ According to evidence, FIB-4 does not perform accurately in some cases, like younger patients (under 35 years) and morbidly obese adults.^34,35^ However it is worth mentioning that FIB-4 can confidently exclude high-grade liver fibrosis in overweight, obese, and excessively obese individuals.^34^ It is suggested that for individuals under 35, noninvasive evaluations besides FIB-4 should be applied for estimating liver fibrosis (e.g. elastography).^24^ Our study’s mean age exceeded 60 years, and the average BMI was below 30, mitigating these potential limitations. In patients with COVID-19, the FIB-4 level was associated with SARS-COV-2 plasma RNA level and monocyte-related cytokine levels.^13^ Our results showed that FIB-4 is firmly linked with the clinical outcomes of COVID-19 such as lower oxygen saturation and higher CT-score, suggesting that the direct effect of SARS-CoV-2 may be a more probable mediator than pre-existing liver dysfunction. FIB-4 is usually used to anticipate the requirement for mechanical ventilation or ICU admission.^27^ The index incorporates age and aspartate transaminase, both significantly related to liver fibrosis,^10,36^ and extensively associated with poor COVID-19 outcomes such as death.^37,38^ Among the components of the FIB-4 index, ALT, AST, and age were significantly higher in the deceased group. The inclusion of age in the FIB-4 formula, unlike other non-invasive fibrosis markers such as the AST/platelet ratio index (APRI), potentially enhances its predictive capacity.^39^ Recognizing that FIB-4 can be elevated due to factors unrelated to liver disease, we excluded patients with diabetes to enhance the specificity of our findings. This approach aimed to isolate the relationship between FIB-4 and COVID-19 outcomes more accurately.

 Based on the evidence, severe acute respiratory syndrome and inflammatory reactions can cause skeletal muscle injury and, a greater right heart pressure. These factors may contribute to elevated FIB-4 indices through multiple mechanisms. Firstly, muscle injury may lead to an increase in AST levels, directly affecting the FIB-4 calculation. Secondly, there is a relationship between COVID-19 and an increase in the right cardiac pressure in recent studies, which is believed to lead to liver congestion, injury, and fibrosis.^40,41^ Thirdly, direct virological impacts may explain the persistently elevated FIB-4 levels observed in patients with severe disease or those who passed away.^39^ Pathological findings in previous studies also showed early evidence of liver fibrosis such as portal fibrosis is common in patients who are infected with COVID-19. One of them also revealed that in 68% of samples, portal dilation, activation of Kupfer cells, and detection of coronavirus in the portal system were identified.^42,43^ Our findings support previous studies indicating that patients with a history of CVD, kidney disease, and cancer are at higher risk of developing severe forms of COVID-19.^44^ Furthermore, we observed significantly higher levels of urea and creatinine in patients who died from COVID-19, consistent with other studies that have identified these parameters as predictive of COVID-19 mortality.^45^ In line with our results, previous studies have reported that lymphopenia, thrombocytopenia, and low albumin levels are common poor prognostic indicators in patients with FIB-4 indices higher than 2.67. These findings suggest that the FIB-4 index may serve as a composite marker reflecting both hepatic and systemic pathophysiological processes in severe COVID-19.^46,47^

 This study has some limitations. First, socioeconomic factors including education, income, and occupational status can affect mortality and access to clinical care. Unfortunately, we did not include these factors in our analysis. Second, to further confirm that elevated FIB-4 is due to liver fibrosis, elastography or liver biopsy would be warranted, which were not feasible in our study in the setting of COVID-19.

 In conclusion, the FIB-4 index is an easily calculable marker derived from routine laboratory tests, which demonstrates potential as a prognostic factor for COVID-19 severity and mortality. This study suggests that the FIB-4 index could serve as a valuable tool for risk stratification in hospitalized COVID-19 patients, potentially facilitating rapid intervention and treatment prioritization for high-risk individuals. The index’s primary advantage lies in its simplicity and accessibility, allowing for quick assessment without the need for advanced imaging techniques. It is suggested to measure FIB-4 at admission for early identification of high-risk patients with severe COVID-19.
